# Prediction of structural features and application to outer membrane protein identification

**DOI:** 10.1038/srep11586

**Published:** 2015-06-24

**Authors:** Renxiang Yan, Xiaofeng Wang, Lanqing Huang, Feidi Yan, Xiaoyu Xue, Weiwen Cai

**Affiliations:** 1 Institute of Applied Genomics, School of Biological Sciences and Engineering, Fuzhou University, Fuzhou 350108, China; 2 College of Mathematics and Computer Sciences, Shanxi Normal University, Linfen, 041004, China

## Abstract

Protein three-dimensional (3D) structures provide insightful information in many fields of biology. One-dimensional properties derived from 3D structures such as secondary structure, residue solvent accessibility, residue depth and backbone torsion angles are helpful to protein function prediction, fold recognition and *ab initio* folding. Here, we predict various structural features with the assistance of neural network learning. Based on an independent test dataset, protein secondary structure prediction generates an overall Q_3_ accuracy of ~80%. Meanwhile, the prediction of relative solvent accessibility obtains the highest mean absolute error of 0.164, and prediction of residue depth achieves the lowest mean absolute error of 0.062. We further improve the outer membrane protein identification by including the predicted structural features in a scoring function using a simple profile-to-profile alignment. The results demonstrate that the accuracy of outer membrane protein identification can be improved by ~3% at a 1% false positive level when structural features are incorporated. Finally, our methods are available as two convenient and easy-to-use programs. One is PSSM-2-Features for predicting secondary structure, relative solvent accessibility, residue depth and backbone torsion angles, the other is PPA-OMP for identifying outer membrane proteins from proteomes.

Proteins act alone or in complexes to perform a wide range of cellular functions in diverse biological processes, including catalyzing reactions^1^, replicating DNA^2^, and transporting molecules^3^. Most proteins naturally fold into native 3D structures which contain vital clues to their biological functions at both molecular and cellular levels. Thus, extensive efforts have been devoted to obtaining the protein 3D structures over the past decades, resulting in a rapidly increasing number of experimentally determined protein structures in recent years. As of January 2015, there exist over 100,000 structures in the Protein Data Bank (PDB)^4^ database which provides crucial information for the development of new bioinformatics tools, such as structure-seeded binding site prediction^5^, fold recognition^6^ and fragment-based *ab initio* folding^7^. In general, various properties can be derived from PDB structures and used to represent the characteristics of sequence environments. Protein secondary structure may be one of the most common elements of 3D structures. Some other one-dimensional structural properties such as relative residue solvent accessibility (RSA), residue depth (RD) and backbone torsion angles (BTA) are also widely explored. The RSA value of an amino acid measures to what extent the amino acid is accessible to the solvent (usually water) surrounding a protein. However, when RSAs are zeros or near zeros, the knowledge of RSA cannot provide information about the structural arrangements of buried residues^8^. As an alternative, RD which refers to the distance from the amino acid to the molecular surface can be viewed as a complementary measure. The BTA of proteins involves the backbone atoms C′-N-C_α_-C′, which is called Phi and N-C_α_-C′-N, which is called Psi. Protein 3D structures are largely determined by the Phi and Psi angles, which provide very helpful information for protein structure determination.

For structurally known proteins, these properties can be directly obtained from PDB coordinates using computational programs. The STRIDE^9^ and DSSP^10^, as examples, are designed to obtain protein secondary structure and solvent accessibility area of PDB structures. Some elegant bioinformatics tools have also been developed to predict these features directly from protein sequences. For instance, Psipred^11^ is a widely used tool to predict protein secondary structure from sequence profiles with a Q_3_ accuracy of approximately 80%. SPINE-X^12^ is a novel method for RSA and BTA prediction through a two-layer neural network. In general, these structural features, even if approximately or by prediction, are still useful for protein structure prediction and function annotations. In fact, most state-of-the-art protein prediction methods use one or some structural features to improve performance. In this work, we develop algorithms to predict SS, RSA, RD and BTA with the assistance of neural network training. We apply the predicted structures to outer membrane protein (OMP) identification from proteome wide sequences. OMPs, which have been found to play diverse functional roles, are frequently found in the outer membranes of gram-negative bacteria, mitochondria and chloroplasts. Due to their functional importance, correct identification of OMPs from high throughput sequences is of value for proteome annotation and drug design^13^. During the past decades, OMP identification techniques have received considerable attention and a series of novel methods (e.g., TMBETA-NET^14^, PRED-TMBB^15^, and HHomp^16^) has been developed. Machine learning methods were also used in OMP identification^17^,^18^. One successful tool is HHomp. To predict whether a query sequence is an OMP, HHomp builds a profile HMM for a query sequence and compares it with an OMP database by HMM-HMM matching.

Previously, we developed SSEA-OMP^19^ for OMP identification and Trans-OMP^20^ for locating the transmembrane regions of OMPs. In SSEA-OMP, secondary structure element alignment (SSEA) is used to align an OMP and a non-OMP databases. The query sequence is judged whether it is OMP or not by the top alignments. In Trans-OMP, transmembrane regions of OMPs are predicted by combining the position- and composition-based features of sequence profiles.

Although many methods have been proposed, the performance of OMP identification is not yet very satisfactory and the requirements for new algorithms are still desirable, especially in the current post-genomic era. Here, we develop a novel method to identify OMPs. We construct sequence profiles (e.g., PSSM) by searching query sequences against the NCBI NR database via Position-Specific Iterated BLAST^21^ (PSI-BLAST). The obtained PSSM profiles are then fed into trained neural networks to generate predicted structural properties. The query sequences are compared with OMPs by using profile-to-profile alignment with a scoring function incorporating PSSM profiles and predicted structural features. Finally, the query sequences are judged to be OMPs or not by the significances of the alignment scores. We will introduce the details of our algorithms step-by-step in the following sections.

## Results

### A pipeline of our methods

**Figure 1:**
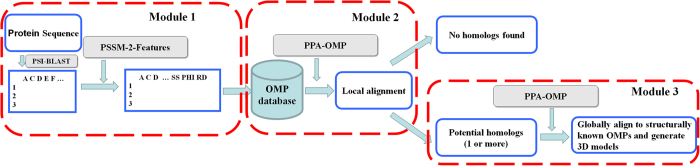
A pipeline of our methods. The pipeline consists of three modules, prediction of structural features, identification of OMPs, and modeling of 3D structures for potential OMPs. First, a target protein is iteratively threaded through the local NCBI NR database for three iterations to generate sequence profiles. Profiles are then fed into the trained neural networks to predict structurally features. Second, the target protein is searched against an OMP sequence database by using PPA-OMP with a scoring function incorporating sequence profiles and predicted structural features. The target protein is judged to be an OMP or not by the significance of the top alignment. Third, the target protein is searched against a structurally known OMP database by PPA-OMP program if the target protein is predicted to be an OMP. The 3D structural models of the target are built using the alignment by PPA-OMP with the assistance of MODELLER^23^ program. Because PPA-OMP is used to search a sequence database and a structurally known database in this pipeline, PPA-OMP is used twice in this flow chart.

A pipeline of our methods was constructed and is clearly presented in Fig. 1. The pipeline consists of three modules: (1) prediction of structural features, (2) identification of OMPs, and (3) modeling of 3D structures for potential OMPs. In the first step, a query sequence is threaded by PSI-BLAST through the NCBI NR database for three iterations with an e-value threshold of 0.001 to generate sequence profiles. Then, structural features can be generated by PSSM-2-Features with the sequence profiles provided as input. In the second step, PPA-OMP local alignment method^22^ is used to search the query sequence against an OMP sequence database, and the query sequence is determined to be an OMP or not by top alignments. Finally, the query sequence is searched against a structurally known OMP database. The 3D models for the query sequence are built using PPA-OMP alignment with the assistance of MODELLER^23^ if it is predicted to be an OMP. It should be pointed out here that global alignment is used to align as many residues as possible in the 3D model building.

### Comparison of protein secondary structures calculated by different methods

**Figure 2:**
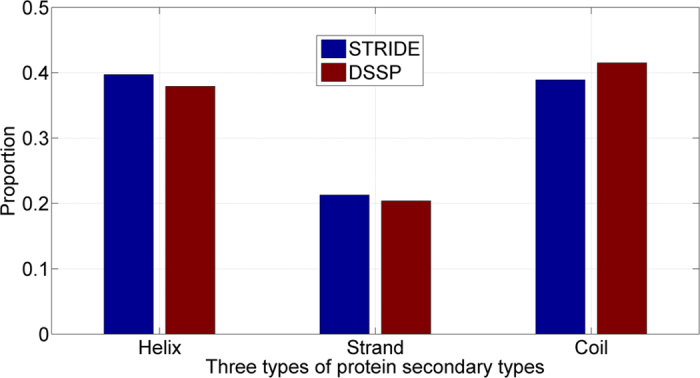
DSSP and STRIDE for assignment of protein secondary structure on SCOPe_TEST1073 dataset.

Protein secondary structures were derived from PDB structures. Nevertheless, defining secondary structures from PDB structures is not an exact process due to the fact that different programs have their own definitions. In fact, different programs have their strengths in assigning α-helix, β-strand or coil. Therefore, using different programs to derive the secondary structures in the benchmark dataset always has a certain level of bias in the results. The contents of secondary structures on SCOPe_TEST1073 by the DSSP and STRIDE programs were summarized in Fig. 2. As shown the Figure, the most proportion of assignments was coil by the two programs. STRIDE defined slightly more α-helix and β-strand than that of DSSP (0.397 versus 0.379, 0.214 versus 0.204), while DSSP defined more coil than STRIDE (0.415 versus 0.388). Overall, the two programs had 94.7% agreement in their assignments.

### Overall performance of PSSM-2-Features

#### Protein secondary structure prediction

Our method (PSSM-2-Features) was trained on PDB_TRAIN6675 dataset and tested on SCOPe_TEST1073 dataset. Table 1 shows the results of protein secondary structure prediction on the SCOPe_TEST1073 dataset and cross-validation result on PDB_CS6001 dataet. We relied mainly on the SCOPe_TEST1073 dataset to assess different methods.

Among the four measures (Q_3_, Q_H_, Q_E_ and Q_C_), Q_3_ is the most comprehensive parameter to assess the performance of secondary structure prediction. The Q_3_ accuracy of PSSM-2-Features is slightly lower than 80%. When protein secondary structure was assigned by STRIDE, the Q_H_, Q_E_ and Q_C_ values of PSSM-2-Features are 0.869, 0.728 and 0.764, respectively. Similar results were obtained when protein secondary structure was assigned by DSSP.

It should be noted that overfeeding of models is avoided by removing similar sequences from training datasets. For example, protein sequences in the PDB_TRAIN6675 dataset were not similar to the sequenes in the SCOPe_TEST1073 dataset at the sequence level (BLAST e-value > 0.001). Similarly, the identity between any two sequences is lower than 30% in PDB_CS6001 dataset.

#### Phi angle, relative solvent accessibility and residue depth prediction

**Figure 3:**
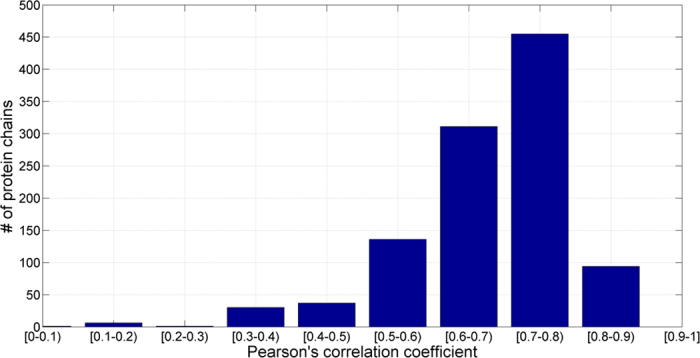
Number of proteins as a function of Pearson’s correlation coefficient (Pcc) for RSA on SCOPe_TEST1073 dataset.

**Figure 4:**
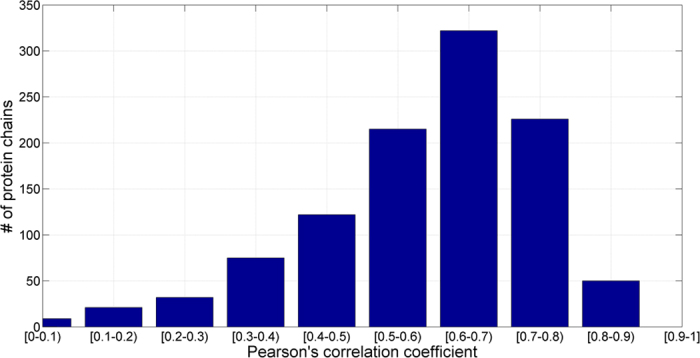
Number of proteins as a function of Pearson’s correlation coefficient (Pcc) for RD on SCOPe_TEST1073 dataset.

Table 2 shows the input features and the optimized window sizes for each structural property in the PSSM-2-Features. The fitness of amino acids in three types of secondary structures is given in Table S1 (supplementary file 1) and used in protein secondary structure prediction of the PSSM-2-Features. The mean absolute errors (MAE) of RSA, RD and Phi predictions were summarized in Table 3. Of the four structural features, RD gave the lowest error value (MAE = 0.062). RSA was the most difficult to be predicted and we obtained a MAE value of 0.164 on SCOPe_TEST1073 dataset. This result may suggest that solvent accessibility is probably less conserved than other properties (e.g., secondary structure) in the protein families, which is consistent with that reported by ROST and Sander^24^. On the other hand, the Pcc scores of RD, Phi and RSA predictions are 0.597, 0.546 and 0.690 on SCOPe_TEST1073 dataset. It is interesting to learn that the Pcc value of RSA is higher than that by RD and Phi although the MAE value of RSA is not better than them. To further investigate the results, the Pcc scores between predicted and actual properties (i.e., RSA and RD) were calculated. The distributions of the Pcc scores for the proteins on the SCOPe_TEST1073 dataset for RSA and RD are shown in Figs 3 and 4, respectively. Overall speaking, the results by our predictors are relatively accurate. The reasons of our algorithm’s effectiveness relying on the factors as: (1) the input features (e.g., PSSM) are informative, and (2) parameters of neural networks4 are highly optimized. Here, we can learn that the structural features are strongly related to evolutionary information (i.e., PSSM profile). Furthermore, we used SS_RI measure (i.e., Eq 12) to estimate the reliability of protein secondary structure prediction for each residue. In our benchmark result, if the score of SS_RI > 0.35, it yields a predicted result with a false positive rate of less than 1%.

### Comparison with state-of-the-art methods

#### Protein secondary structure prediction

PSSpred, Psipred, SABLE and SPINE-X for protein secondary structure prediction were installed in our local computers and test proteins were directly fed into them. Interestingly, all methods tested here are NN-based predictors. Again, we relied mainly on the SCOPe_TEST1073 dataset to assess different methods. When protein secondary structures were assigned by the STRIDE program, PSSpred, Psipred and SPINE-X resulted in Q_3_ accuracies greater than 80%. SABLE and PSSM-2-Features generated Q_3_ accuracies lower than 80%. In contrast to other methods, the Q_E_ of the PSSpred is very high (74.6%), and this is probably because the PSSpred used seven neural networks to make a consensus prediction. When protein secondary structures were assigned by the DSSP program, the PSSpred generated a Q_3_ accuracy slightly higher than 80%, while the Q_3_ scores of Psipred, SPINE-X and PSSM-2-Features were slightly lower than 80%. Similarly, the Q_E_ of the PSSpred is the highest (75.1%) compared with other three methods. It is quite clear that the benchmark results were slightly different when different programs were used to derive protein secondary structure. Similar results were obtained when different methods were tested on PDB_CS6001 dataset.

The Q_E_ scores of the Psipred, the SPINE-X and PSSM-2-Features are relatively lower when compared with Q_H_ and Q_C_ and this can be ascribed to the fact that the formation of β-strand is strongly influenced by long-range interactions^25^, which is hard to be predicted. In 1998, Gromiha and Selvaraj reported a similar result and they found the prediction of all-α proteins was better than that of all-β proteins^26^.

### *Phi angle,* relative solvent accessibility and residue depth prediction

SPINE-X can also be used to predict phi angle. As reported by Singh *et al.*^27^, SPINE-X is one of the top methods for phi angle prediction. Here, SPINE-X results in a MEA value of 0.072 and a Pcc value of 0.550. The performance of PSSM-2-Features for phi angle prediction is slightly worse (MAE = 0.082 and Pcc = 0.546).

Meanwhile, the RSA prediction by SPINE-X is MAE of 0.168 and Pcc of 0.673. The performance of PSSM-2-Features for RSA prediction got similar result (MAE = 0.164 and Pcc = 0.690).

As to the RD prediction, we can not directly compare it with other methods due to the unavailability of their standalone programs and web servers^8^,^28^,^29^. Here, we compared their performance using the data reported in the literatures. Yuan-Wang method^8^ reported to be a MAE value of 0.600 and a Pcc value of 0.650. RDpred^28^ method yielded a MAE value of 0.558 and a Pcc value of 0.670. Prodepth^29^ method generated a Pcc value of 0.710. PSSM-2-Features generated a MAE value of 0.062 and a Pcc value of 0.597 on SCOPe_TEST1073 dataset and a MAE value of 0.083 and a Pcc value of 0.553 on PDB_CS6001 dataet. The performance of PSSM-2-Features on RD prediction can be regarded as worse than other methods.

#### RSA versus RD

**Figure 5:**
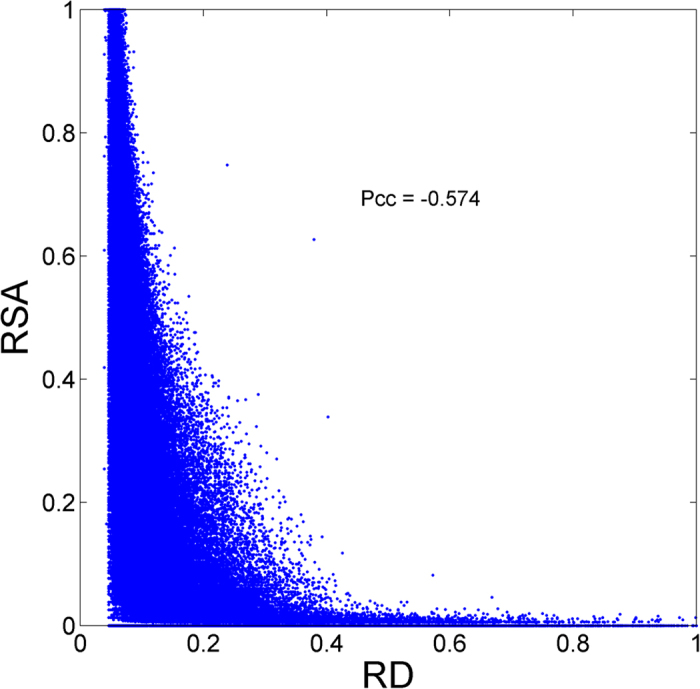
Relationship between RSA and RD on SCOPe_TEST1073 dataset.

We also investigated the correlation between RSA and RD (Fig. 5). RSA measures to what extent an amino acid is accessible to a solvent while RD measures how deeply a given residue is buried. It is not surprising to learn that the Pcc score between RSA and RD is −0.574, which suggests RSA is negatively correlated and complementary with RD.

Some structural features are correlated to a lesser degree, such as SS and RSA. Therefore, it is reasonable that the performance of RSA prediction can be slightly improved by using secondary structure based on this context.

### Benchmark of outer membrane protein identification

**Figure 6:**
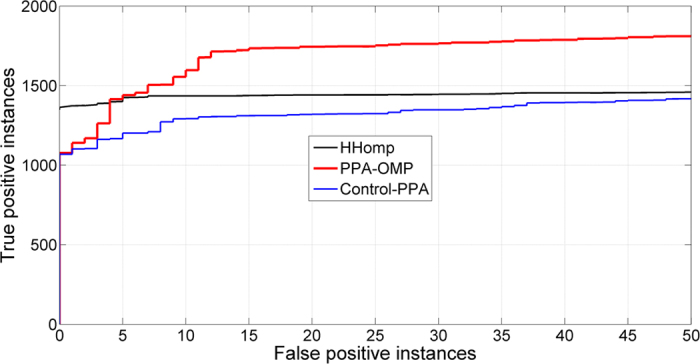
ROC curves of different OMP discrimination methods assessed on R-dataset.

We used the same library and test dataset (i.e., R-dataset) as HHomp to assess our OMP identification method. The performance of OMP identification was compared via ROC analysis. We paid more attention to the performance at <1% (e.g., 50 false positive instances in the R dataset), which is considered a critical threshold in practical applications. As can be seen from Fig. 6 and Table 4 PPA-OMP correctly recognizes 1,314 OMPs before including the first 5 false positives, and, HHomp can detect 1,400 OMPs at the same level. At a 1% false positive rate control, PPA-OMP can correctly recognize 1,741 OMPs, and the number is slightly higher than that identified by HHomp (1,459 OMPs). The RSA, RD and Phi-based terms are informative. This can be clearly demonstrated by a 3% lower sensitivity when these terms were removed from the PPA-OMP method (i.e., Control-PPA). In other words, the Control-PPA method was constructed by using sequence profiles and secondary structure terms. The significance of PPA-OMP alignment scores can also be calculated from the R-dataset. It is estimated that a predicted result is at less than the 1% and 5% false positive rates if the alignment scores are higher than 20 and 15, respectively.

### Benchmark experiment on β-class globular proteins

Since all β-class globular proteins and OMPs share similar 3D structures, it is very necessary to benchmark the performance of PPA-OMP for excluding globular proteins. Here, we randomly selected one protein from each family from the SCOPe_40% dataset. Thus, we compiled a dataset called Beta-G822, which contains 822 all β-class globular proteins. These 822 β-class proteins were directly fed into PPA-OMP, which was constructed using 496 consensus OMPs as the library. There are 34 of these 822 β-class proteins that were predicted to be OMP with scores higher than 99% confident level. This prediction accuracy is very high (95.8%). This result may be attributed to the fact that all β-class globular proteins and OMPs were grouped into different homologous families although they share similar 3D structures. For example, OMPs were grouped into f.5 fold (Outer membrane efflux proteins (OEP)), f.4.2 superfamily (Outer membrane phospholipase A (OMPLA)), f.4.1.2 family (Outer membrane enzyme PagP), etc., while all β-class globular proteins were grouped into b class (i.e., b.*.*.*, where * is a wild symbol). Meanwhile, PPA-OMP is a profile-to-profile alignment method and it can discriminate different protein families. PPA-OMP therefore can accurately exclude β-class globular proteins from OMPs. The prediction results for these proteins are publicly available at http://genomics.fzu.edu.cn/OMP/benchmarks/globular_beta_proteins.tar.bz2.

### Protein structure prediction of OMPs

The algorithms described in this work were seamlessly incorporated into our OMP prediction web server (http://genomics.fzu.edu.cn/OMP/). The web server can accept a single protein, either in plain text or in FASTA format. A multiple FASTA formatted input is also acceptable. The number of proteins is up to 50 in each multiple FASTA input. To build 3D structures of potential OMPs, we compiled a library (http://genomics.fzu.edu.cn/OMP/3DLibrary/) consisting of 154 structurally known protein chains. Each target protein, either assumed or predicted to be an OMP, is threaded through the library with the PPA-OMP alignment algorithm. The final models are built by the alignments between target and identified templates with the assistance of MODELLER^23^. The generated models are reliable only if the query is an OMP. The performance of PPA-OMP is further exemplified in the protein structure prediction of 2O5PA (Figure S1 in supplementary file 2). Although both 2O5PA and 1XKWA are from *Pseudomonas aeruginosa* and are structural homologs, they share a weak sequence similarity. When we searched 2O5PA against the sequences of the OMP library using PPA-OMP, 1XKWA was one of top hits (other top templates were closely homologous proteins). The model built by using the 1XKWA was RMSD of 2.79 Å and TM-score value of 0.798. As reported by Xu and Zhang^30^, the model is reliable if the TM-score value is higher than 0.5. Therefore, the model for 2O5PA by PPA-OMP is high-quality and can be used for further analysis. There are only a few non-redundant structurally known OMPs (~30)^16^,^20^,^31^, and we therefore did not benchmark the performance of protein structure prediction of the PPA-OMP using large scale datasets.

### Proteome-wide OMP identification in *E. coli*

We utilized the complete proteome of *E. coli* to test the performance of our method in a real application. In our previous work^20^, we collected a known OMP dataset consisting of 122 proteins from the *E. coli* proteome by retrieving the annotations from NCBI, PSORTdb^32^, OMPdb^33^ databases and via SPARK-X^34^ fold recognition tool. In this work, the 4,126 sequences of *E. coli* were directly fed into PPA-OMP and 111 proteins were predicted to be potential OMPs with a false positive rate control of 1%. There were 76 out of the 111 proteins in the known *E. coli* OMP dataset. Therefore, these 76 predicted OMPs should be regarded as true positives with high confidence. Details of the 76 identified OMPs and their predicted 3D models are available at http://genomics.fzu.edu.cn/OMP/attachments/. Six computational algorithms (i.e., SOSUI, amino acid, dipeptide, motif, SVM-based methods and a method called “New approach”) were used in TMBETA-GENOME (http://tmbeta-genome.cbrc.jp/annotation/) to annotate OMPs. Here, we assumed a protein is predicted to be OMP with high confidence if at least five computational methods of TMBETA-GENOME predict it as OMP. And we obtained 182 such proteins from TMBETA-GENOME for *E. coli* K12 proteome (http://genomics.fzu.edu.cn/OMP/benchmarks/TMBETA-GENOME_182_OMPs.txt. Interestingly, 70 out of these 76 proteins are included in those 182 proteins predicted by TMBETA-GENOME database.

In the remaining 35 proteins, there exist 9 proteins whose subcellular localizations are annotated as ‘unknown’ or ‘this protein may have multiple localization sites’ in the PSORTdb^32^ database. It should be clearly pointed out that some of these 9 proteins may be OMPs that are not experimentally identified yet. To validate the real types of them may need experimental work or further bioinformatics analysis. The other remaining 26 hits are clearly annotated as non-OMPs based on subcellular localization information in the PSORTdb database, suggesting that they are very likely to be false positives.

In fact, it is estimated that 96 ~ 98% protein sequences in the *E. coli* proteome are non-OMPs^31^,^35^, and it is therefore reasonable to have 30 ~ 40 false positives at a 1% false positive rate.

## Discussions

Taken together, we can clearly draw the following conclusions: (1) accurate predictors for protein structural properties, including SS, RSA, RD and Phi, can be built by combining neural network and effective features, (2) these predicted structural features can be applied to improve the identification of OMPs. In this work, we proved these two conclusions by developing two computational tools, namely PSSM-2-Features and PPA-OMP. PSSM-2-Features was designed for predicting structural features, and PPA-OMP program was for identifying OMPs from proteome wide sequences.

There are two factors, the informative input features and highly optimized neural networks, making the PSSM-2-Features relatively accurate. The effectiveness of OMP identification by PPA-OMP can be attributed to the fact that the accuracies of the developed predictors are relatively high and most β-barrel OMPs are relative by common ancestry^16^.

It should be noted that an obvious drawback of our methods, in contrast to other algorithms that only use the target sequence information, is that predicted structural features may be inaccurate when sequence profiles contain non-homologous proteins. Thus, the performance of both structural feature prediction and OMP identification will be affected in such cases. However, incorporation of predicted structural features will, on average, significantly improve prediction performance, in many realistic applications. Therefore, our web server and programs, most probably, will be useful for researchers in the biological community.

## Materials and Methods

### Data collection and preprocessing

To build reliable models for structural features, it is essential to compile large and non-redundant datasets for training and testing. We collected 6,675 proteins from the PDB^36^ database. The set of the 6,675 proteins was named PDB_TRAIN6675 and used as a training dataset. Furthermore, we compiled a test dataset called SCOPe_TEST1073, which consists of 1,073 proteins from SCOPe (SCOP extended, version 2.03) database^37^, to benchmark the performance of structural feature prediction. These proteins in SCOPe_TEST1073 share low similarity to the proteins in the PDB_TRAIN6675 (BLAST e-value > 0.001). Both PDB_TRAIN6675 and SCOPe_TEST1073 datasets are non-redundant. The PDB_TRAIN6675 was constructed by removing highly similar sequences at a cutoff of 40% identity via CD-HIT^38^. The SCOPe_TEST1073 contains 1,073 superfamilies (i.e., each protein in the dataset is a representative superfamily of SCOPe database). To critically benchmark OMP identification, R-dataset (compiled by Remmert *et al.*^16^), which contains 2,164 OMPs from the TransportDB^39^ database and 5,000 non-OMPs randomly selected from the SCOP^40^ database (version 1.69), was downloaded from ftp://ftp.tuebingen.mpg.de/pub/protevo/HHomp/benchmark/. It should be mentioned that those proteins in the training dataset (i.e., PDB_TRAIN6675) share low similarity to the proteins of both SCOPe_TEST1073 and R-dataset at the sequence level (BLAST e-value > 0.001). We further clustered sequences in PDB_TRAIN6675 dataset with the cutoff of 30% sequence identity via BLASTCluster program in the BLAST package. The highly similar sequences with sequence identity >30% were removed and we obtained 6,001 sequences from PDB_TRAIN6675 dataset in this step. The set of these 6,001 sequences was named PDB_CS6001. The 30-fold cross-validation is used to benchmark prediction on PDB_CS6001 dataset. More details of constructing the datasets are available at supplementary file 3. All datasets and benchmark results in this study can be downloaded at http://genomics.fzu.edu.cn/OMP/benchmarks/.

### Neural network learning

We used neural networks (NNs) to train the predictors for structural features in this work. Prediction performance of NN-based predictors mainly depends upon two factors. One is how much information is contained in input features, the other is the architecture of NNs. A NN is generally composed of three components, i.e., an input layer, one or more hidden layers and an output layer. The training process is to obtain the optimized weights connecting different layers. The training algorithm used in this work was implemented via Encog framework (https://code.google.com/p/encog-java/downloads/list). The learning rate of 0.001 and momentum of 0.85 were found to be effective. The sigmoid activation function (1/(1 + e^−x^)) was applied to hidden and output layers. The architecture and parameters were specifically optimized for each feature predictor. The procedures to develop these algorithms (i.e., predictors for SS, RSA, RD and BTA) were through similar steps: (i) sequence profile generation, (ii) encoding construction and (iii) optimization of parameters of NNs.

### Protein secondary structures

STRIDE^9^ and DSSP^10^ were used to derive protein secondary structure from 3D coordinates. The STRIDE program utilizes both hydrogen bond energy and main chain dihedral angles, to derive secondary structures for structurally known proteins while the DSSP program mainly depending on hydrogen bond energy. The states obtained by STRIDE and DSSP are G (3–10 helix), H (α-helix), I (PI-helix), T (turn), E (extended conformation), B (isolated β-bridge), and C (coil). These seven states are reduced by the following transformations: H, G and I –> H (α-helix), E and B –> E (β-strand), and other states –> C (coil). Different results obtained by STRIDE and DSSP are compared and discussed in this paper. We trained the predictor for secondary structure using a similar way to Psipred. Briefly, two standard feed-forward back-propagation NNs are used. The first NN contains two hidden layers and the second NN has only a single hidden layer. The output layer contains three nodes with each node standing for one secondary structure type. The sigmoid activation function is used and the three secondary structures are therefore encoded as H (0, 0, 1), E (0, 1, 0), and C (1, 0, 0) in the output layer. The generated three secondary structure probabilities of the first neural network are fed into the second neural network that again produces probabilities (i.e., final probabilities).

### Residue solvent accessibility

The relative solvent accessibility (RSA) of an amino acid in a protein measures to the extent of the amino acid accessible to a solvent (usually water) surrounding the protein. In general, hydrophobic amino acids are buried inside the protein while hydrophilic amino acids are on or near the surface. The DSSP program was used to calculate accessible surface areas for all residues in our datasets. The obtained accessible surface areas were then divided by the surface areas of amino acids to get RSA. The surface areas of twenty amino acids were obtained from the reference 41. Because of unusual bond angles, sequence lengths and distorted geometry in real proteins, RSA values can sometimes exceed 100%. We directly set the values to 100% for such cases.

### Residue depth

In contrast to the solvent accessibility, residue depth (RD) measures the degree of inaccessibility of a given residue buried inside a protein. The concept of RD supplements the information provided by RSA. The RD values of proteins were calculated by EDTSurf^42^ program. The values output by EDTSurf lie in [2.8, 9.8], where a higher value corresponds to a deeper region where a residue locate. Using the same method as in FFAS-3D^43^, the RD values were normalized to the range of 0 ~ 1 by an equation as follow


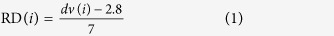


where *dv(i)* is the value output by EDTSurf, and it is absolute residue depth value for the residue *i*. RD(i) is the relative residue depth value for residue i. A model was trained to predict RD scores of proteins.

### Backbone torsion angles

We only trained and used a predictor for Phi angle according to the fact that the predicted result of Psi is not very satisfactory (data not shown). The Phi angles range from −180˚ to 180˚. The angles were transformed to the range of 0˚ to 360˚ by keeping the angles between 0˚ and 180˚ unchanged, and adding 360˚ for angles between −180˚ and 0˚. The angles were then linearly normalized to the range of 0 ~ 1 by dividing by 360˚.

### Fitness of amino acids in secondary structures

We also analyzed the fitness of amino acids in α-helix, β-strand, and coil on PDB_TRAIN6675 dataset and applied it to protein secondary structure prediction. Three probability values for each amino acid appearing in the three types of secondary structures were derived as


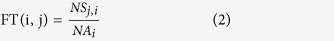


where *NA*_*i*_ is the number of the *i*th residue type in the dataset. *NS*_*j,i*_is the number of the *j*th secondary structure type in the *i*th residue type. *i* is in the range of 1 to 20, representing 20 amino acids, and *j* ranges between 1 and 3, standing for 3 types of secondary structures. The obtained score FT(i,j) is the fitness score between the *i*th residue type and the *j*th secondary structure type. The probabilities of three secondary structure types for any residue sum up to 1.

### Input features

Each target residue is represented by the features of its sequence or structural characteristics. The features for training are usually referred to as input vectors or input features. The construction of input features is mainly based on three observations. First, sequence profiles are important for structurally relative properties. Second, it is helpful to consider adjacent residues of the target residue. Last but not least, some structural properties are somewhat correlated (e.g., SS and RSA). For a query sequence, its sequence profile can be generated by using the PSI-BLAST^21^ to search NCBI NR database for three iterations with an e-value cutoff of 0.001. There are two types of sequence profiles generated using the option ‘-Q’. One is a position specific scoring matrix/profile (PSSM), the other is a position specific frequency matrix/profile (PSFM). Both of the profiles are used in this work. For each residue, a sliding window containing 2n + 1 residue long (i.e., window size = 2n + 1) fragment profiles centered at the target residue is excised from the sequence profile and fed into NNs. For training purpose, all input and output values are scaled to be within the range of 0 to 1. Considering some elements of the PSSM profile are negatives, we directly normalize the values to the range of 0 ~ 1 by the function (1/(1 + e^−*x*^)), where *x* is the element value of the PSSM profile. Meanwhile, we calculate the entropy value of each residue as


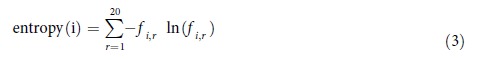


where *f*_*i,r*_ is the frequency of the *r*th residue at position *i* from the PSFM profile. We further propose a conservation score from each residue as


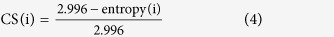


where entropy(i) is the entropy value of residue *i* using Eq. 3 and CS(i) is the conservation score for residue *i*. If a position is very conserved (i.e., only one type of amino acids found in this position), the entropy will be equal to 0. The entropy is close to 2.996 if the residue is highly variable (i.e., the frequencies of twenty amino acids are equal in the position). The CS value lies in (0,1], where a higher score corresponds to a more conserved state for the residue. In addition, an extra unit per amino acid is used to indicate whether the residue spans either the N or C terminus of the protein chain. For region spanning the N or C terminus, the feature values are set to zeros and the value of the additional bit is set to 1, otherwise the value of the bit is set to 0. We carefully selected input features for each specific structural property. For RD and Phi predictions, we used a sliding window containing PSSM profile, PSFM profile, conservation score, and an extra unit per amino acid, indicating whether the residue spans either the N or C terminus of the protein chain. For SS prediction, in addition to the features used by the former two predictors, we further used the fitness of amino acids in SS. For RSA prediction, we used the input features that were used in RD, and, the three probabilities of SS prediction were also employed. It should be clearly noted that the predicted SS used in RSA prediction is not a sliding window, but just the probabilities of three types of SS for the target residue. A sliding window containing secondary structures has also been examined, but no improvement was observed.

### Dynamic programming for outer membrane protein identification

The dynamic programming algorithm was implemented using the procedure described in the book of Durbin *et al.*^44^ (See supplementary file 4 for details). The scoring function used in this work is as


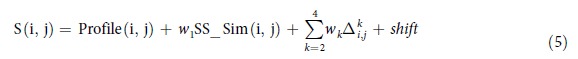


where Profile(i,j) is a simple dot-product profile-to-profile alignment score. SS_Sim(i,j) is a measure of secondary structure similarity. The term 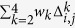 is used to calculate the differences of structural properties between the target and template sequences. The *shift* parameter is introduced to avoid the alignment of unrelated residues in the local regions. We will explain the details of each term in the following sections. The statistical significance of alignment scores is calculated using the same way as our previous work^45^ (See supplementary file 5 for details).

#### Sequence profile

The Profile(i, j) is an evolutionary profiles-based term. The PSSM and PSFM profiles are generated by PSI-BLAST by the option ‘-Q’. The profile similarity score is as


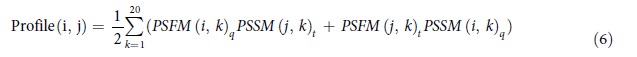


where *PSFM(i, k)*_*q*_ represents the frequency of the *k*th amino acid at the *i*th position of the PSFM profile for a target protein. *PSSM*(*j, k*)_*t*_ denotes the *k*th amino acid at the *j*th position of the PSSM profile for a template. Similarly, *PSFM*(*j, k*)_*t*_ represents the frequency of the *k*th amino acid at the *j*th position of the PSFM profile for the template. *PSSM*(*i, k*)_*q*_ denotes the *k*th amino acid at the *i*th position of the PSSM profile for the target protein.

#### Secondary structure-based term

In our method, the similarity score for each pair of secondary structure profile columns is defined as


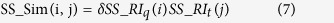


where *SS_RI*_*q*_*(i)* and *SS_RI*_*t*_*(j)*, which are calculated using Eq 17, are the reliabilities of *i*th residue of the target and *j*th residue of a specific template, respectively. 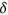 is set to 1 if *i*th and *j*th residues of the target and template proteins are the same type and 0, otherwise.

#### Property-based terms

The symbol 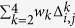 stands for the sum of the differences of three structural features as


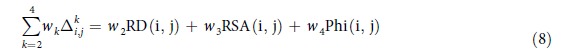


where *w*_*2*_, *w*_*3*_ and *w*_*4*_ are weights to sum these terms. RD(i, j), RSA(i, j) and Phi(i, j) are RD-, RSA- and Phi-based terms. The calculations of them are as


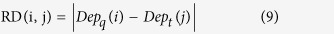



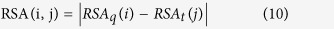



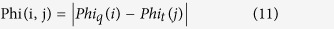


The values of gap opening, gap extension, *w*_*1*,_ *w*_*2*_, *w*_*3*_, *w*_*4*_, and *shift* were obtained by maximizing of the sequence alignments to structural alignments^46^ of all-to-all pair-wises for the 23 structurally known OMPs, which were selected by Remmert *et al.*^16^. Finally, the values of gap opening, gap extension, *w*_*1*,_ *w*_*2*_, *w*_*3*_, *w*_*4*_, and *shift* were set to −7.0, −0.54, 0.65, 1.0, 1.0, 1.0, and 0.76, respectively.

### Outer membrane protein identification

To identify OMPs, we used the developed profile-to-profile alignment method (Eq. 9). We built an OMP library. Here, the OMP database, which was originally compiled by Remmert *et al.*^16^ and derived from 23 structurally known OMPs, was downloaded from ftp://toolkit.genzentrum.lmu.de/pub/HHomp/db/HHompDB_1.0.hhm. There exist 496 consensus sequences in this database. We extracted the 496 consensus sequences from the database and PSI-BLASTed them against the NCBI NR database for three iterations to generate new sequence profiles. The predicted structural features can be generated using the sequence profiles. For a given test protein, we search it against a database consisting of the 496 sequence profiles from these consensus sequences through our profile-to-profile alignment method. The query protein will be determined whether it is an OMP or not by the statistical significances of alignment scores (See our previous work^45^ or supplementary file 5 for calculation of significant scores). The procedure is carried out on all proteins of the R-dataset.

### Performance assessment measure

Q_3_, Q_H_, Q_E_ and Q_C_ are utilized to measure the performance of protein secondary structure prediction. The Q_3_ score is the sum of correctly predicted residue states divided by the total number of residues. Other three measures Q_H_, Q_E_ and Q_C_, which describe the fractions of correctly predicted residues out of the total numbers of residues in α-helix, β-strand and coil, are also used. Furthermore, we also use an equation similar to that proposed by Rost and Sander^47^ to calculate the position-specific reliability index of prediction for each residue as


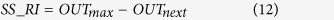


where *OUT*_*max*_ is the output node of the neural network with the highest value, and *OUT*_*next*_ is the second highest value.

As to assessing the performance of RSA, RD and Phi predictions, we use the mean absolute error (MAE), which is a common quantity used to measure how close predictions are to the final outcomes. The MAE is given by


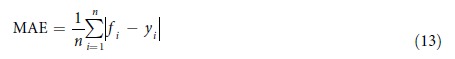


where *f*_*i*_ is the prediction score, *y*_*i*_ is the true value and *n* is the number of residues. On the other hand, prediction performance of RSA, RD and Phi is also measured by the correlation between predicted and actual values using Pearson’s correlation coefficient (Pcc). The Calculation of Pearson’s correlation coefficient is available a supplementary file 6.

The performance of OMP identification can be quantified by ROC curve. By taking false positive rate (instances) as x axis, and true positive rate (instances) as y axis, all the data pairs corresponding to all possible thresholds of prediction scores will make a ROC curve.

## Additional Information

**How to cite this article**: Yan, R. *et al.* Prediction of structural features and application to outer membrane protein identification. *Sci. Rep.* **5**, 11586; doi: 10.1038/srep11586 (2015).

## Supplementary Material

Supplementary File 1

Supplementary File 2

Supplementary File 3

Supplementary File 4

Supplementary File 5

Supplementary File 6

## Figures and Tables

**Table 1:** Comparison of protein secondary structure prediction performance.

**Secondary structure assigned by STRIDE**				
	**Q**_**3**_	**Q**_**H**_	**Q**_**E**_	**Q**_**C**_
PSSpred^a^	0.813	0.876	0.746	0.786
Psipred^a^	0.800	0.813	0.711	0.835
SPINE-X^a^	0.801	0.882	0.695	0.777
SABLE^a^	0.783	0.823	0.662	0.809
PSSM-2-Features^a^	0.798	0.869	0.728	0.764
PSSpred^b^	0.804	0.836	0.727	0.818
Psipred^b^	0.798	0.788	0.699	0.863
SPINE-X^b^	0.800	0.873	0.679	0.801
SABLE^b^	0.786	0.817	0.665	0.826
PSSM-2-Features^b^	0.787	0.853	0.669	0.792
Secondary structure assigned by DSSP				
	Q_3_	Q_H_	Q_E_	Q_C_
PSSpred^a^	0.801	0.877	0.759	0.751
Psipred^a^	0.799	0.824	0.726	0.812
SPINE-X^a^	0.788	0.881	0.707	0.743
SABLE^a^	0.780	0.832	0.677	0.785
PSSM-2-Features^a^	0.793	0.833	0.710	0.799
PSSpred^b^	0.793	0.835	0.738	0.786
Psipred^b^	0.799	0.799	0.714	0.843
SPINE-X^b^	0.788	0.871	0.689	0.768
SABLE^b^	0.786	0.826	0.678	0.805
PSSM-2-Features^b^	0.780	0.831	0.663	0.796

^a^The results here were tested on an independent dataset (i.e., SCOPe_TEST1073).

^b^The results here were tested based on cross-validation on PDB_CS6001 dataset.

**Table 2:** Input features and optimized window sizes for the training of structural properties.

	**Window size**	**# of hidden layers**^a^	**# of NNs**	**Input features**^b^
SS	15	2,1	2	PSSM, PSFM, CS, FT
RD	17	1,1	2	PSSM, PSFM, CS
Phi	17	1	1	PSSM, PSFM, CS
RSA	21	1,1	2	SS, PSSM, PSFM, CS

^a^There are one or two numbers in the column of number of hidden layers. If there are two numbers in, the two numbers are nodes in the first and second networks. Generally speaking, we use the second neural network to refine the prediction by the first neural network.

^b^PSSM, PSFM, FT and CS stand for position-specific scoring matrix, position-specific frequency matrix, amino acid’s fitness score to secondary structure and conservation score, respectively.

**Table 3:** The mean absolute error (MAE) and Pearson’s correlation coefficient (Pcc) of various structural properties.

Property	MAE	Pcc
SPINE-X Phi^a^	0.072	0.550
PSSM-2-Features Phi^a^	0.082	0.546
SPINE-X RSA^a^	0.168	0.673
PSSM-2-Features RSA^a^	0.164	0.690
PSSM-2-Features RD^a^	0.062	0.597
SPINE-X Phi^b^	0.074	0.549
PSSM-2-Features Phi^b^	0.082	0.546
SPINE-X RSA^b^	0.153	0.688
PSSM-2-Features RSA^b^	0.164	0.690
PSSM-2-Features RD^b^	0.083	0.553

^a^The results here were tested on the SCOPe_TEST1073 dataset.

^b^The results here were tested based on cross-validation on PDB_CS6001 dataset.

**Table 4:** Comparison of receiver operator characteristics table for different methods.

**Receiver operator characteristics (≤50 false positives**^a^)										
	**5**^b^	**10**	**15**	**20**	**25**	**30**	**35**	**40**	**45**	**50**
HHomp^c^	1400	1435	1437	1441	1442	1445	1449	1454	1455	1459
PPA-OMP^c^	1314	1389	1452	1541	1564	1634	1667	1706	1728	1741
Control-PPA^c^	1166	1291	1310	1319	1323	1346	1362	1393	1404	1417

^a^Here, false positives correspond to those non-OMPs that are predicted as OMPs.

^b^The numbers in this line show various thresholds of false positives.

^c^The numbers in these lines correspond to true positives that can be identified by methods tested here.
