# Supplementary material for: Prediction of structural features and application to outer membrane protein identification
Source: Sci Rep. 2015 Jun 24;5:11586. doi: 10.1038/srep11586 (PMC4478468; doi:10.1038/srep11586)
Supplement: Supplementary File 1 [file srep11586-s1.doc]

**Supplementary file 1: Fitness of amino acids in protein secondary structures**

**Table S1. Fitness of amino acids in protein** secondary structures

|  | H^a^ | E^a^ | C^a^ |
| --- | --- | --- | --- |
| A | 0.492 | 0.166 | 0.341 |
| C | 0.288 | 0.278 | 0.434 |
| D | 0.281 | 0.125 | 0.594 |
| E | 0.468 | 0.150 | 0.382 |
| F | 0.354 | 0.299 | 0.348 |
| G | 0.158 | 0.144 | 0.699 |
| H | 0.305 | 0.217 | 0.478 |
| I | 0.371 | 0.357 | 0.272 |
| K | 0.404 | 0.174 | 0.422 |
| L | 0.465 | 0.230 | 0.305 |
| M | 0.438 | 0.212 | 0.350 |
| N | 0.280 | 0.135 | 0.584 |
| P | 0.146 | 0.098 | 0.756 |
| Q | 0.456 | 0.166 | 0.378 |
| R | 0.420 | 0.198 | 0.382 |
| S | 0.281 | 0.184 | 0.535 |
| T | 0.271 | 0.261 | 0.468 |
| V | 0.318 | 0.402 | 0.280 |
| W | 0.364 | 0.279 | 0.357 |
| Y | 0.337 | 0.306 | 0.357 |

^a^For protein secondary structure, H represents a helix element, E denotes a strand element, and C stands for a coil element.
