# Supplementary material for: Prediction of structural features and application to outer membrane protein identification
Source: Sci Rep. 2015 Jun 24;5:11586. doi: 10.1038/srep11586 (PMC4478468; doi:10.1038/srep11586)
Supplement: Supplementary File 2 [file srep11586-s2.doc]

**Supplementary file 2: Cartoon representation of 3D structure prediction by the PPA-OMP with the assistance of MODELLER program**


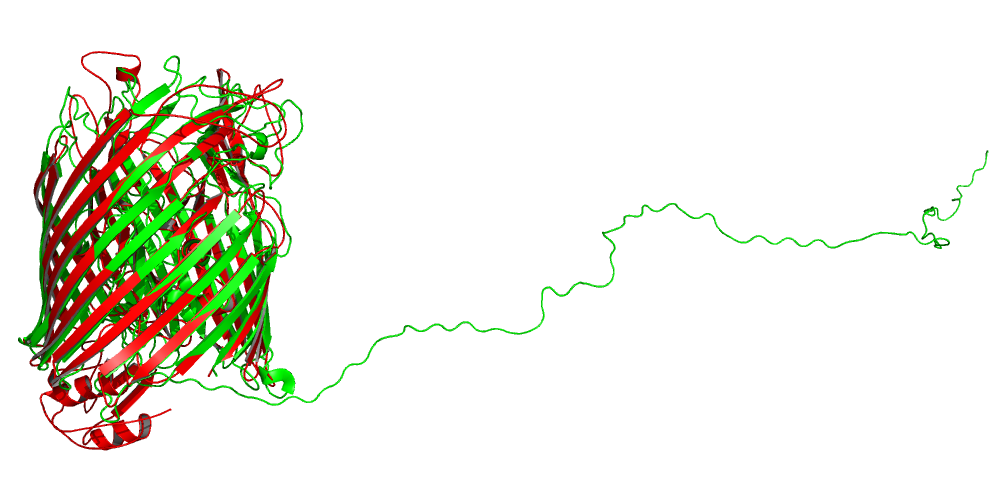


**Figure S1. Cartoon representation of 3D structure prediction by the PPA-OMP with the assistance of MODELLER** [^27^](#_ENREF_27) **program.** The structural alignment between 2O5PA native structure (red) and the model (green) is presented. The PPA-OMP uses 1XKWA as a template. The identity of global sequence alignment between 2O5PA and 1XKWA is 25%. Therefore, the 1XKWA can be regarded as a remote homolog of 2O5PA. The RMSD and TM-score values for the model are 2.79 Å and 0.798, suggesting the model is reliable. The 1~60 residues of 2O5PA were aligned to a region of gaps, and the model therefore contains a starting coil region.
