# Supplementary material for: Prediction of structural features and application to outer membrane protein identification
Source: Sci Rep. 2015 Jun 24;5:11586. doi: 10.1038/srep11586 (PMC4478468; doi:10.1038/srep11586)
Supplement: Supplementary File 3 [file srep11586-s3.doc]

**Supplementary file 3: Collection and preprocessing of datasets**


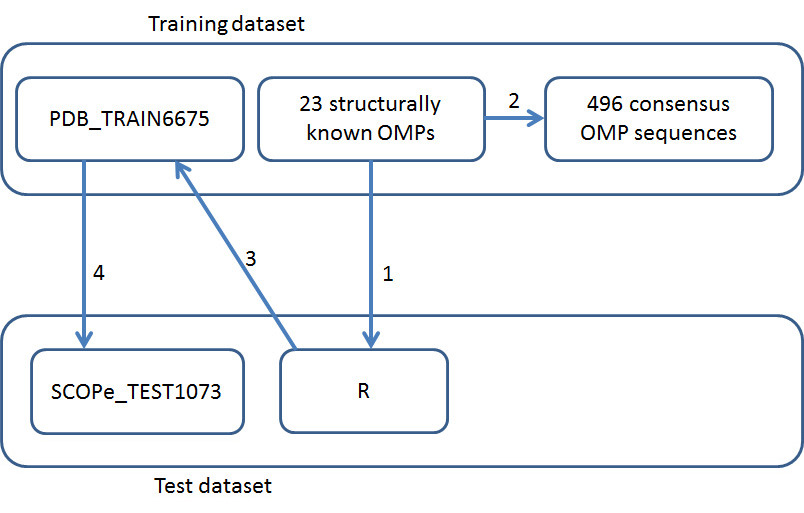


**Figure S2. The flow chart of data collection and preprocessing.** The numbers (i.e., 1, 2, 3, and 4) denote the order of constructing and collecting the datasets.

The construction of datasets was started by collecting R-dataset, which was originally compiled by Remmert *et al*[^1^](#_ENREF_1). The sequences of R-dataset show low sequence similarity with the 23 structurally known OMP (BLAST e-value>0.01). The 23 structurally known OMPs were used to derive the 496 consensus OMP sequences. The details of R-dataset, 23 structurally known OMP, and consensus OMP sequences can refer to the work of HHOMP[^1^](#_ENREF_1) by Remmert *et al*.

In this work, we collected R-dataset, 23 structurally known OMP, and the 496 consensus OMP sequences. Then, SCOPe_40% database with a total of 11,999 sequences was downloaded from SCOPe^2^ database (version 2.03), in which the sequence identity among the proteins is equal to or less than 40%. These 11,999 sequences were searched against the R-dataset. We collected those proteins that were not similar to R-dataset at the sequence level. Meanwhile, we only randomly selected one protein from one family from the remaining proteins, and we obtained 1,073 proteins. The set of these 1,073 proteins was named SCOPe_TEST1073 dataset. Then, we collected 6,675 proteins from Protein Data Bank[^3^](#_ENREF_3) (PDB) database to train our models. These 6,675 proteins were non-redundant (sequence identity among the proteins is equal to or less than 40%) and were not similar to the proteins of SCOPe_TEST1073 dataset.

Here, PDB_TRAIN6675 and SCOPe_TEST1073 sets were used as a training and an independent datasets, respectively. Cross-validation of the training dataset is also very important to assess the performance of methods. We further clustered sequences in PDB_TRAIN6675 dataset with a cutoff of 30% sequence identity via BLASTCluster program in the BLAST[^4^](#_ENREF_4) package. We obtained 6,001 sequences from PDB_TRAIN6675 dataset in this step. The 30-fold cross-validation is used here. The PDB_TRAIN6675 dataset is randomly partitioned into 30 nearly equal size folds (i.e., subsamples). Of the 30 folds, a single fold is retained as the validation data for model testing, and the remaining 29 folds are used as training data. The cross-validation process is then repeated 30 times (the folds), with each of the 30 folds used exactly once as the validation data.

Furthermore, we randomly selected one protein from each family from SCOPe_40% dataset. Thus, we compiled a dataset called Beta-G822, which contains 822 β-class proteins. These 822 β-class proteins were used to assess the performance of PPA-OMP for excluding β-class globular proteins.

All datasets and benchmark results are publicly available at <http://genomics.fzu.edu.cn/OMP/benchmarks/>.

**References**

1. Remmert, M., Linke, D., Lupas, A.N. & Soding, J. HHomp--prediction and classification of outer membrane proteins. *Nucleic acids research* **37**, W446-451 (2009).

2. Fox, N.K., Brenner, S.E. & Chandonia, J.M. SCOPe: Structural Classification of Proteins--extended, integrating SCOP and ASTRAL data and classification of new structures. *Nucleic acids research* **42**, D304-309 (2014).

3. Berman, H.M. The Protein Data Bank: a historical perspective. *Acta crystallographica* **64**, 88-95 (2008).

4. Altschul, S.F., Gish, W., Miller, W., Myers, E.W. & Lipman, D.J. Basic local alignment search tool. *Journal of molecular biology* **215**, 403-410 (1990).
