# Supplementary material for: Prediction of structural features and application to outer membrane protein identification
Source: Sci Rep. 2015 Jun 24;5:11586. doi: 10.1038/srep11586 (PMC4478468; doi:10.1038/srep11586)
Supplement: Supplementary File 4 [file srep11586-s4.doc]

**Supplementary file 4: Implementation of dynamic programming algorithm**

We used the dynamic programming algorithm to combine predicted structural terms by the trained neural networks for OMP identification. The algorithm was implemented using the procedure described in the book of Durbin *et al*[^1^](#_ENREF_1) as follows

$\mathrm{M}\left(\mathrm{i},\mathrm{j}\right)=\max\left\{\begin{matrix} \mathrm{M}\left(\mathrm{i}-1,\mathrm{j}-1\right)+\mathrm{S}\left(\mathrm{i},\mathrm{j}\right) \\ \mathrm{I}_{\mathrm{x}}\left(\mathrm{i}-1,\mathrm{j}-1\right)+\mathrm{S}\left(\mathrm{i},\mathrm{j}\right) \\ \mathrm{I}_{\mathrm{y}}\left(\mathrm{i}-1,\mathrm{j}-1\right)+\mathrm{S}\left(\mathrm{i},\mathrm{j}\right) \end{matrix}\right.$ (1)

$\mathrm{I}_{\mathrm{x}}\left(\mathrm{i},\mathrm{j}\right)=\text{max}\left\{\begin{matrix} \mathrm{M}\left(\mathrm{i}-1,\mathrm{j}\right)-o \\ \mathrm{I}_{\mathrm{x}}\left(\mathrm{i}-1,\mathrm{j}\right)-e \end{matrix}\right.$ (2)

$\mathrm{I}_{\mathrm{y}}\left(\mathrm{i},\mathrm{j}\right)=\text{max}\left\{\begin{matrix} \mathrm{M}\left(\mathrm{i},\mathrm{j}-1\right)-o \\ \mathrm{I}_{\mathrm{y}}\left(\mathrm{i},\mathrm{j}-1\right)-e \end{matrix}\right.$ (3)

where *x* and *y* correspond to two protein sequences being aligned. *i* and *j* are the position indexes of residues. *o* and *e* are gap opening and extension penalties. M(i,j) is the best score up to the position (*i*, *j*) in the scoring matrix of dynamic programming assuming that *x_i_* is aligned to *y_j_*. I_x_(i, j) is the best score assuming that *x_i_* is aligned to a gap and I_y_(i, j) is the best score given that *y_j_* is aligned to a gap. S(i, j) is the scoring function of aligning *i*th position of the *x* sequence to the *j*th position of the *y* sequence.

**References**

1. Richard Durbin, S.R.E., Anders Krogh, Graeme Mitchison. Biological Sequence Analysis: Probabilistic Models of Proteins and Nucleic Acids*. CAMBRIDGE UNIVERSITY PRES***S Chapter** 2, 30-31 (1998).
