# Supplementary material for: Prediction of structural features and application to outer membrane protein identification
Source: Sci Rep. 2015 Jun 24;5:11586. doi: 10.1038/srep11586 (PMC4478468; doi:10.1038/srep11586)
Supplement: Supplementary File 5 [file srep11586-s5.doc]

**Supplementary file 5**: **Statistical significance of alignment scores**

We used the same way as that in our earlier work[^1^](#_ENREF_1) to calculate the significances of alignment scores. Briefly, we used 1,187 proteins, called SCOPe_1187 dataset, which are from SCOPe database, to calculate the mean and standard deviation of random scores. ZScore can be calculated by an equation as

$\text{ZScore}=\frac{raw-mean}{std}$ (1)

where *raw* score is the alignment score of the target to a specific template. *mean* and *std* are the average score and standard deviation of the target sequence aligning to those 1,187 proteins in the SCOPe_1187 dataset. There exist two ZScores for any pair of target-template alignments. One is calculated for the target sequence (i.e., *ZScore_q_*), the other is for a specific template (i.e., *ZScore_t_*). The calibrated ZScore is calculated using a way similar to FFAS-3D[^2^](#_ENREF_2) as

$\text{ZScore}_{\text{final}}=0.5\left(ZScore_{q}+ZScore_{t}\right)$ (2)

where ZScore_final_ is the average of *ZScore_q_* and *ZScore_t_* as the finally calibrated score, which is symmetrical with respect to two aligned proteins.

**References**

1. Yan, R.*, et al.* GPCRserver: an accurate and novel G protein-coupled receptor predictor. *Molecular bioSystems*  (2014).

2. Xu, D., Jaroszewski, L., Li, Z. & Godzik, A. FFAS-3D: improving fold recognition by including optimized structural features and template re-ranking. *Bioinformatics (Oxford, England)* **30**, 660-667 (2014).
