# Supplementary material for: Prediction of structural features and application to outer membrane protein identification
Source: Sci Rep. 2015 Jun 24;5:11586. doi: 10.1038/srep11586 (PMC4478468; doi:10.1038/srep11586)
Supplement: Supplementary File 6 [file srep11586-s6.doc]

**Supplementary file 6: Calculation of Pearson’s correlation coefficient (Pcc)**

Pearson’s correlation coefficient (Pcc) is calculated as

$\text{Pcc}=\frac{n\sum_{i=1}^{n} X_{i}Y_{i}-\sum_{i=1}^{n} X_{i}\sum_{i=1}^{n} Y_{i}}{\sqrt{n\sum_{i=1}^{n} X_{i}^{2}\text{-}\sum_{i=1}^{n} X_{i}\sum_{i=1}^{n} X_{i}}\sqrt{n\sum_{i=1}^{n} Y_{i}^{2}\text{-}\sum_{i=1}^{n} Y_{i}\sum_{i=1}^{n} Y_{i}}}$ (1)

where *n* is the number of sample pairs. *X_i_* and *Y_i_* are predicted and the real values for observation *i* of a specific structural property, respectively. The first and last residues of each protein are not used in the calculation according to the fact that most scores of these positions are set using default values.
